# Thermally Stable Nanotwins: New Heights for Cu Mechanics

**DOI:** 10.1002/advs.202203544

**Published:** 2022-10-26

**Authors:** Thomas Edward James Edwards, Nadia Rohbeck, Emese Huszár, Keith Thomas, Barbara Putz, Mikhail Nikolayevich Polyakov, Xavier Maeder, Laszlo Pethö, Johann Michler

**Affiliations:** ^1^ Empa Swiss Federal Laboratories for Materials Science and Technology Laboratory for Mechanics of Materials and Nanostructures Feuerwerkerstrasse 39 Thun 3602 Switzerland; ^2^ Department of Materials Science Montanuniversität Leoben Franz Josef‐Strasse 18 Leoben 8700 Austria

**Keywords:** copper, high temperature creep, mechanical strength, nanoparticles, nanotwins

## Abstract

Nanocrystalline and nanotwinned materials achieve exceptional strengths through small grain sizes. Due to large areas of crystal interfaces, they are highly susceptible to grain growth and creep deformation, even at ambient temperatures. Here, ultrahigh strength nanotwinned copper microstructures have been stabilized against high temperature exposure while largely retaining electrical conductivity. By incorporating less than 1 vol% insoluble tungsten nanoparticles by a novel hybrid deposition method, both the ease of formation and the high temperature stability of nanotwins are dramatically enhanced up to at least 400 °C. By avoiding grain coarsening, improved high temperature creep properties arise as the coherent twin boundaries are poor diffusion paths, while some size‐based nanotwin strengthening is retained. Such microstructures hold promise for more robust microchip interconnects and stronger electric motor components.

## Graphical abstract

Ultrahigh strength nanotwinned copper microstructures have been stabilized against high temperature exposure while largely retaining electrical conductivity. Adding less than 1 vol% W nanoparticles increases hot hardness and mitigates creep by stabilizing nanotwinned Cu up to 400 °C.

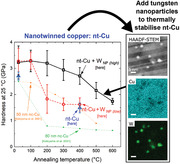

## 1 Introduction

Refining grain size down to the nanometer scale is an effective way to enhance the strength of metals while retaining ductility. Strength increases inversely proportional to the square‐root of grain size: the Hall–Petch relationship.^[^ ^1^ ^]^ In a typically soft metal, copper, a nanohardness beyond 3 GPa is achieved simply by processing to a 10 ± 5 nm grain size.^[^ ^2^ ^]^ Alongside impeding dislocation motion for strength, the inherently large grain boundary (GB) area of nanocrystalline metals unfortunately also facilitates creep mechanisms at low homologous temperatures compared with coarse‐grained counterparts.^[^ ^3^ ^]^ Even at ambient temperature, pure nanocrystalline copper (nc‐Cu) can be unstable, undergoing abnormal grain growth,^[^ ^4^ ^]^ which prevents use of Hall–Petch strengthening. Previous attempts at stabilizing nc‐Cu for high temperature strength relied on addition of several percent of a second phase.^[^ ^5^ ^]^ A trade‐off ensues against the high thermal and electrical conductivity of pure copper, as in conventional alloying of the copper lattice.^[^ ^6^ ^]^ High strength, high conductivity, and thermomechanical fatigue resistance are crucial for reliable copper metallizations in micro‐, nano‐, and flexible electronics, and equally so for enabling first generation hybrid‐electric commercial aircraft motor technology.^[^ ^7^ ^]^ This calls for additional strengthening mechanisms to improve on nc‐Cu.

The high density of twin boundaries (TB) in nanotwinned copper (nt‐Cu) strengthens by Hall–Petch similarly to GBs.^[^ ^8^ ^]^ Compared to random, high angle GBs, twins have low energy boundaries, which thus possess better thermal stability. Their presence can provide some degree of grain size retention.^[^ ^9^ ^]^ However, the thickness of nanotwins themselves increases considerably by ≈200 °C,^[^ ^10^ ^]^ again forfeiting their strengthening contribution.

In this work, we substantially extend the temperature range of effective nanotwin strengthening by adding monodisperse, insoluble second phase nanoparticles to a highly refined nt‐Cu microstructure. Recently, preliminary results showed this approach is effective at thermally stabilising equiaxed 28 nm nc‐Cu at 500 °C.^[^ ^11^ ^]^ Here, the idea is twofold. First, the nanoparticles facilitate nanotwin formation during initial deposition, avoiding the need for interrupted sputtering.^[^ ^9^, ^12^ ^]^ Second, the nanoparticles stabilize microstructure by pinning TBs and GBs at elevated temperatures, and may directly limit dislocation movement by Orowan bowing. The low nanoparticle content (< 1 vol%) retains good electrical conductivity.

## 2 Results

**Figure 1:**
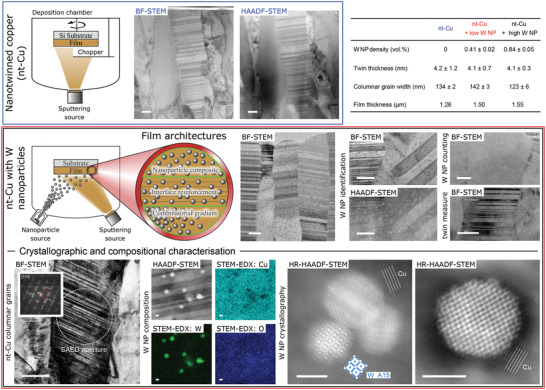
Nanotwinned Cu films with and without embedded W nanoparticles. Scanning transmission electron microscopy (STEM) images of cross‐sectional lift outs of nt‐Cu films with and without W NP—see embedded table for color scheme. Pure nt‐Cu is produced by interrupted DC magnetron sputtering using a chopper system, while W NP are codeposited from a nanoparticle source with a high power impulse sputtered Cu matrix (see Methods, adapted from^[^ ^11^ ^]^). This leads to several opportunities for W NP concentration gradients laterally and through‐thickness: those exploited here are outlined in green. Columnar nanotwinned grains (confirmed by selected area electron diffraction, SAED) dominate the microstructure for all W NP densities. The W NP are darker dots in the bright field (BF) images (0.84 vol% W NP shown here); they can be confirmed as W NP in the high angle annular dark field (HAADF) images (light dots, by atomic number contrast), rather than crystal defects of the Cu matrix (deposition or deformation‐induced). STEM‐EDX chemical mapping supports this, noting the absence of a measurable oxide shell to the NP, while high resolution imaging of the embedded W NP identifies at least one allotrope of W: the A15 phase—see Supporting Information S1 for additional characterization of W NP. Variations in the incidence angle allowed contrast of the twin boundaries to be extinguished to facilitate W NP counting: see Supporting Information S2, also for details on error analysis. The as‐deposited twin thickness and column widths are considerably similar between film compositions, as desired for ease of mechanical property analysis; error values of measured are the standard error in the mean. All scale bars are 50 nm long, except those for STEM‐EDX and HR‐STEM, which are 2 nm long.

Three copper films were fabricated by physical vapour deposition (PVD). All were densely nanotwinned and contained 0.84 ± 0.05, 0.41 ± 0.02, and 0 vol% tungsten nanoparticles (W NP)—named “high,” “low,” and “without.” The W NP were 4.0 ± 0.7 nm in diameter,^[^ ^11^ ^]^ incoherent with the Cu matrix and generated by terminated gas condensation (**Figure** **1**; and Supporting Information S1 for further analysis). This corresponds to an average W NP spacing within Cu slip planes^[^ ^13^ ^]^ of 28 and 42 nm, respectively, for the two nanoparticle‐containing films. Deposition conditions of Cu were selected per W NP condition to achieve identical texture and microstructural dimensions of the Cu matrix; this allows individual strengthening mechanisms to be effectively isolated. All films consisted of columnar grains with strong 111 axial texture; for most grains, densely spaced Σ3 coherent TBs lay approximately parallel to the substrate. The measurements of particle densities, matrix, and film dimensions by transmission electron microscopy (TEM) (Figure 1) yielded average column diameters close to ≈130 nm and average TB spacings of ≈4.1 nm for all three films.

The electrical resistivity of the pure nt‐Cu was determined by the van der Pauw method to be 2.85 × 10^−8^ Ω m at room temperature, which is 70% higher than that of annealed, coarse grained, oxygen‐free high‐conductivity copper (OFHC),^[^ ^14^ ^]^ and its conductivity is 60% of the international annealed copper standard (IACS^[^ ^6^ ^]^). Addition of W nanoparticles raised the resistivity less than 10%, to 2.98 × 10^‐8^ and 3.26 × 10^‐8^ Ω m, for 0.41 and 0.84 vol% W NP, respectively, i.e., an increase by linear fitting of 4.7 × 10^‐9^ Ω m (vol% W NP)^‐1^. Literature supported analysis (Supporting Information S3) confirms increased resistivity of nt‐Cu above OFHC to be due to high Cu–Cu TB and GB areas, and these boundaries remain the dominant factor in raising the resistivity even when W NP are added below 1 vol%. Despite these higher resistivities, such results are promising as nt‐Cu has previously been shown to achieve 97% IACS given larger TB spacings (20 nm^[^ ^10a^ ^]^) —W NP addition as here would hence little deteriorate this. One should also consider that the resistivity of equiaxed nc‐Cu is substantially higher—1.85 × 10^−7^ Ω m for 15 nm grains,^[^ ^10a^ ^]^ i.e., at least five times higher than any film here, for a room temperature hardness within 10% of the present nt‐Cu.

**Figure 2:**
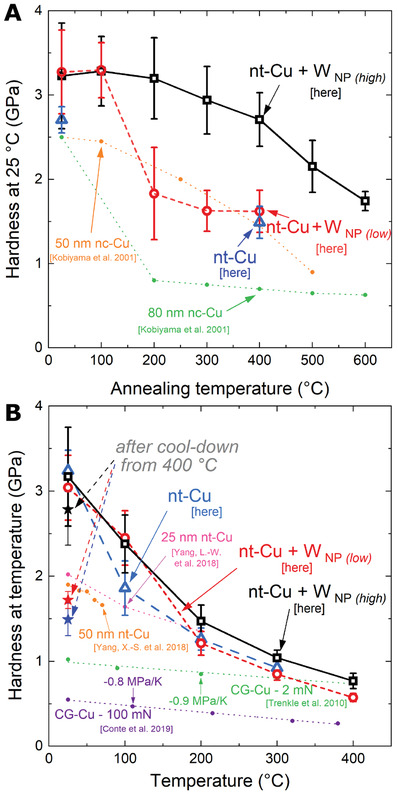
Nanoindentation hardness of nt‐Cu films following heat treatments, and at high temperature. A) Room temperature hardness following successive 1 h anneals, demonstrating the mechanical stability of the nt‐Cu + 0.84 vol% W NP (“high”) condition. The pure nt‐Cu reference underwent all annealing steps, omitting selected intermediate hardness measurements. B) Hardness upon monotonic temperatures increase: a considerable drop occurs for all film compositions in 25–200 °C. Literature data from^[^ ^15^ ^]^ is designated as CG: coarse grained (linear datafits given in B) ); nt: nanotwinned; nc: nanocrystalline; lengths indicate initial TB spacing and grain diameter, respectively. nc‐Cu data represent the extrema of performance previously identified.^[^ ^15a^ ^]^ Error bars relate the standard deviation of measured values—mainly from roughness of the as‐deposited films, which is somewhat reduced upon annealing. All indents were performed to 1 mN at a 0.2 s^−1^ indentation strain rate. Star‐shaped datapoints B) represent room temperature hardness upon cool‐down from the maximum temperature of 400 °C. Anneals at 500 and 600 °C were performed on separate samples as single temperature anneals.

Hardness was determined by Berkovich nanoindentation. All samples exhibited a high hardness for Cu at room temperature (**Figure** **2**B), with values between 3.1 and 3.3 GPa. Room temperature hardness was measured after annealing the same test‐pieces for 1 h at successively higher temperatures (Figure 2A) in ultrahigh vacuum to avoid oxidation. X‐ray diffractometry (XRD) was also performed at each step to capture texture evolution. Both W NP‐containing samples retained initial hardness after annealing at 100 °C (Figure 2A); however, after 200 °C, the hardness of the lower concentration film dropped by 44%, while the hardness of the high W NP film was unchanged. After annealing at 300 and 400 °C, hardness further decreased, more gradually, for both W NP films, ending at 84%, 50%, and 55% of initial hardness for high, low, and without W NP, respectively. Changes in elastic modulus were minimal within error (Supporting Information S4). The initial Cu 111 out‐of‐plane texture, seen as strong (111)_Cu_ XRD peaks (Supporting Information S5), remains until 200 °C; only relaxation of in‐plane compressive stress from film deposition is noted. While the high W NP film displays no further textural changes up to 400 °C, the lower content film exhibits significant changes from 300 °C: an increasingly 200 out‐of‐plane texture develops, which is not seen in the nt‐Cu film without W NP after 400 °C. Additional annealing of the 0.84 vol% W NP film at 500 and 600 °C led to a steeper reduction in hardness than below 400 °C, to 54% of the initial hardness after annealing at 600 °C—where crystal orientation mapping (Supporting Information S6) determined 200 out‐of‐plane texture, whereas the nanotwinned microstructure after 500 °C was only partially coarsened.

Additionally, hot hardness, up to 400 °C (i.e., 0.5 homologous), was measured using nanoindentation in situ in a scanning electron microscope, on as‐deposited samples (Figure 2B) with the same cumulative thermal exposure approach as the annealing study. The hardness of all films dropped drastically with increasing temperature. The nt‐Cu reference (without W NP condition) loses hardness fastest, falling below 2 GPa by 100 °C, whereas both W NP containing films are at ≈2.4 GPa. Above this temperature, the high W NP film consistently outperforms the other two, and all nt‐Cu literature with higher TB spacings.^[^ ^15b,c^ ^]^ With a 0.77 GPa hardness for high W NP at 400 °C, this only equates to coarse‐grained copper measured with the same order of nanoindentation size effects (Trenkle et al.,^[^ ^15d^ ^]^ replotted in Figure 2B). This is consistent with the 43% tensile strength loss from 25 to 180 °C seen for TB spacings down to 5 nm.^[^ ^10b^ ^]^ From 200 °C, the low W NP film matches, within error, the hardness of the reference film. Hardness after cool‐down was consistent with the annealing study.

Therefore, the presence of a sufficient density, 0.84 vol%, of 4 nm diameter W nanoparticles led to both texture stability, where a lower (or null) W NP content did not, as well as an ensuing hardness invariance even after exposure to 400 °C, and a 34% higher hardness at 400 °C than the low W NP alternative.

**Figure 3:**
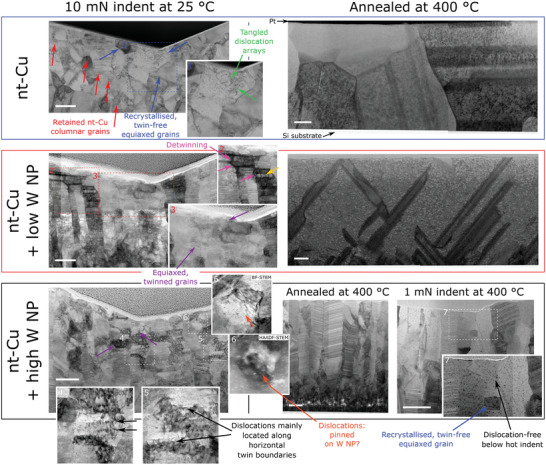
Microstructural conditions following extensive deformation, and after exposure to 400 °C. Bright field scanning TEM images (stitched) of cross‐sections of deep, room temperature Berkovich indents of as‐deposited films—through the indent center, parallel to an indenter edge—showing a loss of the columnar grained, nanotwinned structure below the indent apex in the nt‐Cu reference film. Beyond these equiaxed twin‐free grains (blue arrows) containing tangled dislocation arrays (green, detail 1), columnar nt‐Cu remains, laterally and below (red). Both W nanoparticle‐containing samples retain the nanotwins, even within the few approximately equiaxed grains arising at the apex region (e.g., purple, detail 3). Dislocations accumulate here along twin interfaces, details 4&5, with detwinning (pink) prominent near the indenter‐film interface and dislocation tangles (yellow) throughout these remaining thicker twins. In detail 6, curved line features (orange) are suggestive of dislocations bowing between W NP; positive identification of this process is hampered by small particle sizes: HAADF atomic contrast assists differentiation between W NP (bright contrast) and matrix dislocation debris or loops that may all appear as point features in BF‐STEM images. Annealing at 400 °C led to massive grain growth in the “without” and “low” W NP films; grains regularly extend the full film thickness; in contrast, the columnar nt‐Cu microstructure was stable in the “high” W NP film. Note, the 10 mN indents are useful for studying plasticity mechanisms; however, they penetrate beyond one tenth of the film depth: reliable hardness measurements, Figure 2, were hence performed to 1 mN. All scale bars are 200 nm long.

TEM imaging of high W NP nt‐Cu following annealing at 400 °C (**Figure** **3**) showed the columnar grain width grew minorly, from 123 ± 6 nm as‐deposited, to 132 ± 24 nm; the increased spread suggests some abnormal (heterogeneous) grain growth. The average twin thickness evolved from 4.1 ± 0.3 to 4.1 ± 0.5 nm after 400 °C: it was surprisingly stable, explaining the hardness conservation noted earlier. The distribution of W nanoparticles was still uniform, no change in nanoparticle size or coalescence beyond the initial randomness of deposition was noted. In contrast, both the low and without W NP films were extensively modified: grains exceeded 1 µm, i.e., too large for reliable size statistics on cross‐sectional TEM‐liftouts (≈15 µm in length). Additionally, the Cu nanotwins were entirely lost in both conditions; only a handful of coarse growth twins remain, likely developed upon grain coarsening at high temperature. In low W NP nt‐Cu, the distribution and size of W nanoparticles again remained unchanged.

Further, TEM imaging of the nanotwin, nanoparticle and dislocation structures immediately below indents at 25 and 400 °C was performed to better understand the interaction between mechanisms conveying plasticity and the microstructural features. For both temperatures, and both high/low W NP conditions, the nanoparticle concentration in the region 100 nm below the indent apex was seen to have consistently increased, up to 28% (Supporting Information S2) depending on indentation depth, relative to the deformation‐free material at that temperature. This “particle crowding” phenomenon^[^ ^16^ ^]^ upon indentation, with preferential displacement of the Cu matrix away from the film volume below the indenter, is illustrated in greater clarity in Supporting Information S2 on a Cu film with a through‐thickness W NP gradient, which gives a more exaggerated response.

The columnar nt‐Cu structure of the pure reference transforms into ≈150 nm diameter approximately equiaxed grains immediately below deep room temperature indents, forming a horizontal layer a few grains thick. Previous studies on the topic have referred to strain‐induced de‐nanotwinning—a process of TB migration by a debated ledge mechanism,^[^ ^17^ ^]^ eventually leading to twin thickness increase. However, the authors would argue that the present, and certain other reports,^[^ ^18^ ^]^ display grain morphologies characteristic of conventional recrystallization^[^ ^19^ ^]^ to equiaxed grains. Real detwinning is instead observed to have occurred in, e.g., the low W NP film (Figure 3 detail 2), evidenced by large deflections of the columnar GB from the vertical, distinctive of dislocation‐mediated mechanical twinning.^[^ ^20^ ^]^ The TB spacing increases locally from 4 nm initially, to over 40 nm. At greater depths below this indent, the columnar grain structure is unchanged, albeit dislocated. The W NP‐containing samples mainly retain nanotwinned character near the indentation surface—although this is not visible on all grains at a given imaging angle (Supporting Information S6)—while nanotwins are lost in the nt‐Cu reference. This clearly highlights the ease of nanotwin formation and retention with W nanoparticles. Dislocations in the nt‐Cu reference form Taylor networks in the newly‐formed monolithic grains^[^ ^18a^ ^]^ (Figure 3 detail 1). In the W NP‐containing films, dislocations are repeatedly pinned on and extend between TBs with a morphology as previously reported in nt‐Cu^[^ ^21^ ^]^ (Figure 3 details 2, 4&5). In most cases (e.g., Figure 3 detail 6), it is complex to differentiate between pinning on the TBs or on the W NPs due to the finite thickness of the TEM lamella—although some features are suggestive of the latter mechanism.

After 400 °C indentation and further annealing to perform subsequent indents, much of the indentation cavity of the high W NP film was annealed out,^[^ ^22^ ^]^ along with any dislocations not on TBs (Figure 3). Only columnar grains with horizontal nanotwins and W nanoparticles remain, characteristic of the as‐deposited structure, along with few small recrystallized grains.

Finally, it is noted that the nanotwinned microstructure of the high W NP condition was stable at room temperature over a three year period, with twin thickness averaging at 4.1 ± 0.3 nm for each of the datasets from the initial lifted and imaged TEM samples, and those lifted at the end of the study from that same nt‐Cu film stored in air; the columnar grain width measured 122 ± 4 nm after 3 years—unchanged from the initial 123 ± 6 nm.

## 3 Discussion

Twin boundaries are known to be as effective as grain boundaries in increasing the strength of copper,^[^ ^8a^ ^]^ even below the critical length for effective dislocation pile‐up (≈50 nm^[^ ^23^ ^]^). With further refinement of TB spacing, *λ*, under 15 nm, plasticity becomes dominated by dislocation nucleation from the high TB density, rather than limited by the repeated intersection of mobile dislocations with TBs (Hall–Petch).^[^ ^8b^ ^]^ Initially, this was ascribed to an elevated density of pre‐existing TB Shockley partial dislocations per volume;^[^ ^8b^ ^]^ others later pinpointed dislocation nucleation (DN) occurring at the intersection between TBs and GBs through simulation efforts.^[^ ^24^ ^]^ In contrast, softening of nanocrystalline Cu grains below 10 nm^[^ ^8b^ ^]^ is instead due to grain boundary sliding.^[^ ^25^ ^]^ Recent calculations^[^ ^26^ ^]^ indicate the critical twin thickness for maximum strength, *λ*_c_, is dependent on grain size (column diameter), *d*: for *d* ≈ 130 nm here, *λ*_c_  =  4.7 nm. It is in this softening regime that the present ≈4 nm thick twins operate. DN‐based strength is expressed as^[^ ^24^ ^]^

(1) $$\sigma_{\mathrm{DN}}=M\left[\frac{\Delta U}{S^{*}V^{*}}-\frac{\mathrm{kT}}{S^{*}V^{*}}\ln\left(\frac{d\nu_{D}}{\lambda \dot{\varepsilon }}\right)\right]$$

which considers a stress‐biased energy barrier activation rate for dislocation nucleation—each dislocation producing a plastic strain *b*/*λ*, for a Burgers vector length *b*. $\dot{\varepsilon }$ is the strain rate (using the indentation strain rate at maximum depth here), Δ*U* is the activation energy of atomic motion in the Cu lattice (left‐hand athermal term), which temperature *T* modulates, considering *ν*_D_ the Debye frequency, *k* the Boltzmann constant, V* the activation volume, S* describing stress concentration at the TB‐GB intersection (Supporting Information S7) and *M* the Taylor factor (3.06 for FCC here).

W NP containing films can also exhibit Orowan strengthening: to propagate, dislocations must bow outwards when pinned along their line length by nanoparticles. Equation (2) considers the average intersection radius of the particles, *r*_s_, related to the real W NP radius *r* (2 nm) by $r_{s}=\sqrt{2/3}r$, giving a strength increase^[^ ^13^ ^]^

(2) $$\Delta \sigma_{\mathrm{Oro}}=0.4M\frac{Gb_{p}}{\pi \chi }\ln\left(\frac{2r_{s}}{b_{p}}\right){\left(1-\nu \right)}^{-0.5}$$

with *b*_p_ the Burgers vector of a Shockley partial dislocation in Cu, *G* the shear modulus and *ν* the Poisson ratio. The inter‐particle spacing, *χ*, in the dislocation plane is calculated from the volume fraction of particles, *f*, as:^[^ ^13^ ^]^ $\chi =\left(\sqrt{\pi /f}-2\right)r_{s}$. The alternative mechanism of nanoparticle cutting by dislocations requires a higher stress than Orowan bowing according to Ansell–Lenel theory (Supporting Information S7) and is therefore surmized to not be active here: the small spacing of incoherent W NP impedes sufficient stress accumulation by dislocation pile‐up for cutting to occur.

**Figure 4:**
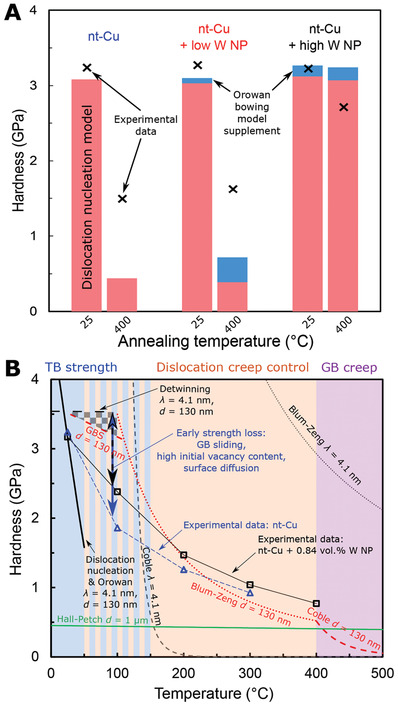
Comparison of experimental data for nt‐Cu + W NP with strengthening and creep models. A) Room temperature hardness as a function of microstructure (as‐deposited and post‐400 °C annealed), using measured lengths and nanoparticle densities (Figures 1 and 3) in the calculations of the quadratically combined dislocation nucleation‐controlled strength (Equation (1)), and the supplemental Orowan bowing contribution (Equation (2)) models. Underestimates of strength of ≈1 µm coarse grain conditions likely result from additional Taylor hardening now active. B) Domains of predominance of strengthening and creep models, based on a simplified nt‐Cu microstructure of *λ* = 4.1 nm TB spacing and *d* = 130 nm diameter columnar grains; this is compared with *d* = 1 µm equiaxed Cu: see Supporting Information S7 for complete curves. The temperature range centred about ≈100 °C where experimental data deviates most strongly from the models is arrowed: a 33–50% lower hardness than model predictions is measured. The gray checkerboard region indicates the range of possible strengths if considering sliding of the columnar grain boundaries (GBS), with a literature‐based^[^ ^31^ ^]^ contribution fraction of 0.6. In B), quadratically combined dislocation nucleation and Orowan bowing strengths (as in A)) are calculated at constant *f* = 0.84 vol% W NP.

Combining strength contributions, nanohardness can be estimated from yield strength with a Tabor coefficient of 3, in a generalized form as $H_{\mathrm{tot}}=3{\left[\sigma_{\mathrm{DN}}^{i}+\Delta \sigma_{\mathrm{Oro}}^{i}\right]}^{1/i}$ where *i* lies anywhere between 1 and 2. Linear addition is inaccurate for composite materials,^[^ ^27^ ^]^ since these strengthening mechanisms do not necessarily operate independently. Here, a quadratic sum is proposed according to.^[^ ^28^ ^]^ Further strengthening mechanisms were considered negligible (Supporting Information S7). Calculated net strengths are compared with experimentally determined values (**Figure** **4**A) for as‐deposited and 400 °C‐annealed conditions, and detailed in (Supporting Information S7). It is evident that the dominant contribution to strength is through grain size refinement—as Hazzledine commented,^[^ ^29^ ^]^ and expanded in,^[^ ^30^ ^]^ particle diameter should drop below 0.8 nm for the Orowan mechanism to outpace the strength benefit of Zener pinning‐decreased grain sizes. In fact, as we calculated on nc‐Cu with W NP,^[^ ^11^ ^]^ the grain size stabilized by standard Zener pinning is 81 and 166 nm for W NP at 0.84 and 0.41 vol%, respectively. The refinement of nanotwins here far exceeds simple Zener pinning.

If one considers an axis along the length of columnar grains, normal to the Si substrate, the average spacing of W NP in the grain projected onto this axis is given by 4/*d* ^2^ *πn*, where *n* is the volumetric number density of particles measured from the TEM images. This spacing is 0.34 and 0.51 nm for the high and low W NP concentrations, respectively—considerably smaller than the 4.1 nm TB spacing. Put differently, this is 12 and 8 W NP on average within each twin, respectively. Hence the W NP density far exceeds the minimum theoretically necessary to nucleate each TB on a W NP—in fact, additional TB nucleation beyond the minimum for a given average spacing is known to occur in nt‐Cu, often reported as loss of TB registry, disregistry, across the grains.^[^ ^18^ ^]^

Microstructural stability is the single most significant factor in ensuring the high strength of Cu against thermal exposure (Figures 3 and 4A). Although nanotwins in pure Cu provide some degree of stability,^[^ ^9^ ^]^ the extent is debated, recently being correlated to initial texture.^[^ ^9b^ ^]^ Broadly speaking, literature and the present study concur that neither pure nc‐Cu^[^ ^11^, ^32^ ^]^ nor nt‐Cu^[^ ^10^ ^]^ is stable at 400 °C (Figure 3). Here, hardness decreases by a factor of two after only 200 °C (Figure 2B): nt‐Cu grains often coarsen to a twin‐free (100) texture in thin films.^[^ ^9b^ ^]^ In fact, the single study on nt‐Cu in contention,^[^ ^9a^ ^]^ reporting some nanotwin stability up to 800 °C, refers to unintended Fe nanoparticles (deposition impurities) visible in a subset of TEM images, that may fortuitously play an equivalent stabilising role to W NP here. In contrast to pure nt‐Cu, the high W NP addition leads to nanotwin retention well beyond a simple Zener pinning law, with better stability than can be expected for these initially over‐refined, homogeneous microstructures:^[^ ^33^ ^]^ no measurable increase in TB spacing is found after heating to 400 °C, within error. One may even speculate that the W NP also lead to regeneration of nanotwins upon strain‐induced dynamic recrystallization of the as‐deposited microstructure (purple arrows, Figure 3). The minor strength loss (≈14%) (Figure 2A) is expected,^[^ ^34^ ^]^ as the solution strengthening high vacancy content of magnetron sputtering is annealed out upon heating above the deposition temperature, 25 °C. Underestimation of the strength of the annealed pure nt‐Cu and low W NP conditions in Figure 4A can be explained by additional Taylor hardening (pinning by dislocation networks) that is now enabled at this coarsened grain size,^[^ ^35^ ^]^ but was not included in the model here.

The high GB area of nanocrystalline metals makes them more susceptible to thermal softening due to easy thermally activated diffusive deformation processes, e.g., creep,^[^ ^3^ ^]^ and GB sliding.^[^ ^36^ ^]^ Coarse‐grained Cu sees a 0.8–0.9 MPa K^−1^ linear decrease in hardness between 25 and 400 °C^[^ ^15d,e^ ^]^ (Figure 2B). For nt‐Cu, the more rapid, nonlinear strength loss with temperature was greater in the without and low W NP films, where the nanotwinned structure was lost (Figure 2B), such that effectively single Cu grains were indented above ≈200 °C. Any strength there could only stem from lattice resistance, Taylor hardening and the ≈1.5 µm film thickness (size‐based strength). Indeed, equal strengths for 0 and 0.41 vol% W NP indicate the bowing of dislocations around the lower W NP content contributes negligible high temperature strength—dislocation climb around such small particles being thermally activated.^[^ ^37^ ^]^

In contrast, the hardness was 34% greater at 400 °C in the high W NP film, and nanotwins remain stable. Nevertheless, the flow stress plunges below the expected temperature dependence of a Hall–Petch‐type strength of the nanotwins. This breakdown is ascribed to the predominance of creep upon heating: for nt‐Cu, two bounding mechanisms are grain boundary diffusion (Coble) creep and Blum–Zeng creep.^[^ ^38^ ^]^ The latter describes dislocation creep in the presence of the high density of high angle GBs characteristic of ultrafine grained (UFG) metals, which affect dislocation abundance.^[^ ^39^ ^]^ At only 0.5*T*_m_, bulk creep (Nabarro–Herring) is negligible.^[^ ^40^ ^]^ Boundary sliding‐based mechanisms are reasoned^[^ ^26^ ^]^ to be substantially repressed in nanotwinned metals by the high density of coherent TBs—therefore predominantly active along the GBs, just 10–15% of the total boundary area or volume here. Both creep mechanisms and GB sliding models are plotted (Figure 4B) along with the temperature‐dependent strengths for Hall–Petch, TB‐GB intersection DN control,^[^ ^24^ ^]^ and twin annihilation by TB migration.^[^ ^26^ ^]^ Further information on calculations and extensive model output is provided (Supporting Information S8).

The direct observation of mechanisms of diffusion‐mediated flow (creep) is notoriously difficult: only a handful of in situ microscopy studies exist and most relate to the special case of irradiation‐induced creep^[^ ^41^ ^]^ where rates are heightened by abundant vacancies generated by in situ ion irradiation. Other studies are limited to low melting point metals like Al.^[^ ^42^ ^]^ For most materials, as here, the analysis of creep mechanisms relies upon the comparison of the temperature‐stress–strain rate relationship of deformation against physics‐based analytical models for specific mechanisms, supported by postmortem observation of deformed microstructures and remnant dislocations. We hence infer (Figure 4B) that due to the high strain rate of indentation (0.2 s^−1^), Coble creep should only become competitive above 400 °C for the range of grain sizes arising (≈130 nm–1 µm). Coble creep theory excessively overestimates the degree of softening, if one considers the TB spacing as the effective grain size for creep (Figure 4B). This plainly demonstrates the creep benefit of the high density of Σ3 coherent TBs in nt‐Cu: coherency avoids rapid boundary diffusion along most crystal interfaces.^[^ ^43^ ^]^ Blum–Zeng creep has a Hall–Petch type dependence of flow stress on grain size; with a 4.1 nm grain size (TB spacing), an unrealistically high flow stress is predicted (Figure 4B). However, for grains sizes of 130 nm and 1 µm, minimum flow stresses are achieved with this mechanism between ≈150 and 400 °C. Our experimental results on the high W NP, permanently nanotwinned film with *d* ≈ 130 nm match the Blum–Zeng creep well above ≈150 °C (Figure 4B). This suggests that, at sufficiently high temperatures, flow is controlled by the size of the nt‐Cu grains, i.e., the density of high‐angle GBs. This would, somewhat counterintuitively, favor smaller nt‐Cu grain sizes for greater strength at temperatures up to 400 °C, at the medium (≈10^−1^ s^−1^) strain rate here. Conversely, to increase strength above 400 °C, grain sizes above 130 nm are desirable. Up to 400 °C, maintaining a nanotwinned microstructure still remains beneficial for creep strength, versus grain coarsening to an equiaxed twin‐free UFG microstructure.

Models and commentary for the without and low W NP conditions are in Supporting Information S8.

**Figure 5:**
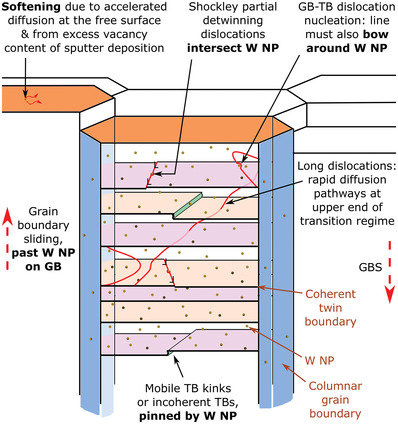
Mechanisms of deformation thought to be operative in the transition regime 50–150 °C, based on existing theories and the present experimental data. Although the detwinning mechanism appears central to controlling deformation in this regime—whether by Shockley partial motion, or migration of coherent TB kinks or incoherent TBs—precocious softening is thought to occur by early activation of creep mechanisms, due to the high vacancy content of sputtered films, and the free surface.^[^ ^8^, ^10^, ^17^, ^24^, ^26^, ^45^ ^]^ W NP are found to be effective in stabilizing the nanotwinned microstructure and slowing deformation across the entire 25–400 °C range studied, as illustrated.^[^ ^13^ ^]^

However, in the range 50–150 °C, all nt‐Cu here were bounded, yet poorly described, by all models. This reinforces recent critical reviews:^[^ ^44^ ^]^ studies of creep behavior in nanotwinned metals are few, and the mechanisms remain poorly understood. This initial rapid strength loss lacks a theory‐based mathematical description, being only described empirically to‐date.^[^ ^15b^ ^]^ It is clear (Figure 4B) that the thermal term of the dislocation nucleation model,^[^ ^24^ ^]^ Equation (1), softens too greatly above room temperature to control flow alone. A recent molecular dynamics simulation study^[^ ^45^ ^]^ found that when *λ* drops to the order of 1 nm, TB migration controls the temperature dependence of flow; such detwinning was indeed observed experimentally here (Figure 3). This mechanism of TB movement leading to twin annihilation presents a considerably more moderate temperature dependence (Figure 4B),^[^ ^26^ ^]^ due to the larger activation volume. The present study on PVD films therefore points toward flow in the 50–150 °C range being controlled by TB migration, combined with additional partial softening by GB sliding^[^ ^26^ ^]^—the complex stress state of nanoindentation also containing a shear component along the columnar GBs. Further early softening here is attributable to accelerated creep at moderate temperatures from the high initial vacancy content of sputtering and the supplementary diffusion afforded by the free surface of indentation. The additional strength retention of the W NP‐containing films in this transitional regime must result from W NP retarding the controlling TB migration mechanism. The specific detwinning process is debated between the passage of Shockley partials along successive (111) planes (∥ TB) of a twin, and the migration of coherent TB kinks^[^ ^10b^ ^]^ or incoherent TBs.^[^ ^17a^ ^]^ Nevertheless, in all cases these dislocations, kinks or boundaries must logically intersect every single W NP within a twin volume to detwin, and they are susceptible to having their motion slowed (Orowan or Zener) by the W NP. This range of mechanisms is illustrated in **Figure** **5**. A hindrance to GB sliding by the W NP that decorate the thermally stable columnar GBs is also surmized.

Such interpretations also put into question whether dislocation nucleation at TB‐GB intersections^[^ ^24^ ^]^ indeed controls strength at room temperature: further cryogenic mechanical testing is required to determine whether detwinning instead better describes the strength evolution of nt‐Cu below 25 °C. Both mechanisms indeed predict similar strengths (both lower than Hall–Petch) at room temperature for the ≈4 nm TB spacing here (Figure 4B). Thus far, cryogenic data are only available for the Hall–Petch strength‐controlled regime: nt‐Cu with ≈50 nm TB spacing.^[^ ^46^ ^]^

## 4 Conclusion

The codeposition of less than 1 vol% of 4 nm tungsten nanoparticles in copper facilitates nanotwin formation, and stabilizes the microstructure against coarsening up to at least 400 °C, hence retaining ≈90% initial hardness after annealing (≈3 GPa). This momentous improvement over coarse‐grained and nanocrystalline alternatives corroborates our preliminary results on nc‐Cu^[^ ^11^ ^]^ and holds promise for applications in microelectronics and electric motors, as it is achieved with only a 9% rise in electrical resistance. At 400 °C, a hot hardness of 0.77 GPa is achieved with W nanoparticles: these are reasoned to resist detwinning processes. Assuming application temperatures below 400 °C, this analysis finds that, quite counterintuitively, a reduction in grain size below the present ≈130 nm is required to further improve high temperature strength—due to the Blum–Zeng creep mechanism describing deformation above 150 °C. Alternative, more conductive nanoparticle elements, such as Ag may lead to an even lesser electrical conductivity drop, if they remain insoluble in the nanotwinned matrix. We expect these conclusions, and our interpretation of the transition regime before standard creep control onsets, to be relevant in understanding the temperature dependence of deformation mechanisms in other nanotwinned metals.^[^ ^47^ ^]^

## 5 Experimental Section

### Deposition of nt‐Cu with W NP

The nt‐Cu films were deposited on Si(100) wafers with 100 nm SiO_2_/100 nm Si_3_N_4_ diffusion barriers by magnetron sputtering in a UHV chamber (M600, Mantis, UK) housing both a magnetron with HiPIMS supply for deposition of the copper matrix from a 50 mm target (99.99%, HMW Hauner, Germany) operating at 100 Hz, for 40 µs, averaging 66 W & 88 mA, and a nanoparticle source (Nanogen 50, Mantis, UK) with a 50 mm tungsten target (99.99%, idem) operating at 52 W & 251 mA. W nanoparticle formation is due to the increased Ar process gas inside the aggregation zone ahead of the sputtered W target, as well as the intermittent contamination of the aggregation volume with air. The latter is a necessary addition to the previous work^[^ ^11^ ^]^ to deposit films with uniform tungsten content along thick cross‐sections (> 1 µm here); without this, nucleation efficiency decreases significantly in under 1 h. Further details are given in Supporting Information S10. It is thought the increased nucleation efficiency after contamination is related to oxidation or nitridation of sputtered W atoms or the W target surface, onto which molecules the metallic nanoparticles can nucleate heterogeneously with greater ease. The deposition at a Cu rate of 0.0675 nm s^−1^ was divided into 13 cycles of 1713 s, between which all process gas flows were halted, along with the power to both magnetrons. Once the 8.5 × 10^−7^ mbar base pressure was achieved, an aggregation zone leak valve opened for 246 s, raising the pressure of the main chamber to 6.6 × 10^−6^ mbar; the pressure of the aggregation zone at this point was approximately ten times higher. Following this, the main chamber was pumped again to the base pressure, before deposition at room temperature under 2.8 × 10^−3^ mbar from 20 & 50 sccm Ar in the main chamber and aggregation zone, respectively; the aggregation zone is cooled to −50 °C to increase W NP nucleation.

The nanoparticle beam produces a circularly‐symmetric deposit on the rotating (5 rpm) substrate, with greatest W NP density on the central axis of the beam, decreasing monotonically outward (lateral combinatorial gradient, Figure 1). Hence, the two nanoparticle densities of the present study were extracted from the same deposition, at the W NP beam center (0.84 vol%) and at a radius of ≈4 cm (0.41 vol%).

To achieve a pure nt‐Cu film with an equal twin thickness and columnar grain diameter as the W NP containing films for direct comparison of strengthening mechanisms, a static chopper system with rotating substrate stage was implemented to interrupt deposition at the correct rate^[^ ^12^ ^]^ for nanotwin thicknesses to match those by W NP cosputtering. This is the PVD process analogous of the pulsed electrodeposition^[^ ^10a^ ^]^ commonly used for nanotwin formation. Operating the Cu magnetron in DC at 200 mA yielded a deposition rate of 0.195 nm s^−1^ during individual expose durations of 6 s: ≈1.2 nm layer^−1^.

### Microstructural Characterization

Films were thermally annealed in the UHV chamber at the above base pressure for 1 h at successively higher temperatures, Figure 2A, with heating and cooling at 150 K h^−1^, together on a single Mo and Ni superalloy holder for nanoindentation and XRD (D8 Discover, Bruker, USA). The latter employed Cu K_*α*1_ (1.54 060 Å) and Cu K_*α*2_ (1.54439 Å) radiations, and *θ*‐2*θ* scanned from 35° to 100° in 0.01° steps, collecting for 1 s at each on 40 kV, 40 mA, with a *θ* offset of 1° from symmetrical diffraction to avoid the Si substrate (400) reflection, and data analysis in DIFFRAC.EVA V5.1 (Bruker, USA).

Samples thinned for electron transparency were extracted from the Cu films as cross‐sectional liftouts along the centers of the Berkovich indents, perpendicular to one of the three edges of the surface triangle, with a preliminary protection by electron, then ion‐deposited Pt. A large thinned width (15–20 µm) by Ga^+^ focussed ion beam (Helios NanoLab 660i, FEI, USA) enabled the undeformed material properties, far from the indents, to be ascertained. Bright field projection (BF‐TEM) transmission electron imaging at 200 kV (Titan Themis G2, FEI, USA) revealed twin spacing, achieved by tilting the sample to align per grain for edge‐on imaging of the TBs. Additional bright field and high angle annular dark field (66–200 mrad) in aberration‐corrected scanning mode (BF‐ & HAADF‐STEM) facilitated the measurement of W NP number densities. To complete number density measurement, film thickness at each imaged location was measured by high resolution SEM based on the STEM probe contamination method. Further description of errors of measurement for error values in Figure 1 is given in Supporting Information S2. High resolution scanning TEM of individual W NP was carried out on highly thinned, low FIB voltage polished specimens (2 kV, 50 pA), while chemical mapping by EDX employed the FEI SuperEDX system. Several elements related to the support structure and holder, and surface contamination, were excluded from the elemental quantization (only used for peak deconvolution): C, N, Al, Ga, Mo, Pt; quantization was based on the L‐lines for Cu and W, and the K‐lines for O. A 5 px local averaging prefilter step was applied to the dataset for Figure 2 before quantization of the 0.5 Å pixel, 5 µs px^−1^ dwell raw acquisition of 900 drift corrected frames. Analysis of W NP‐free subregions of scans of the composite found the W content to be below the measurement threshold of STEM‐EDX.

Alternative conventional chemical composition measurement methods were considered to directly measure the atomic fraction of tungsten in the films. However, although it would be feasible for sufficient material to be dissolved in a liquid solution for the W signal to be within range of detection (given successful delamination of the film), it was considered that the result would not critically improve the understanding of the material system, such as the electrical conductivity of the nt‐Cu matrix. This is because as the W content of the Cu matrix is not directly accessed through this method—nor the O or Cu impurity content of the W NP. Only an average atomic fraction for the entire film is accessed. Hence the volume fraction of nominally insoluble tungsten nanoparticles is the more important value to consider in terms of evaluating the benefits of W NP on mechanical, electrical, and microstructural stability properties.

Electron backscatter diffraction (EBSD) was carried out using a Symmetry camera (Oxford Instruments, UK) on a dual‐beam electron microscope (Lyra 3, Tescan, Czechia) and a 5.1 nA probe of 20 kV electrons. Data were analyzed in OIM Analysis (EDAX, USA) using a single step grain dilation process, without replacement of indexed pixels.

### Mechanical Characterization

Room temperature Berkovich nanoindentation, Figure 2A, of the annealed nt‐Cu films (Ubi, Hysitron Inc., USA) fixed on the permanent Mo holder, with constant test parameters (maximum load 1 mN, 2 s hold, in load control at 0.2 mN s^−1^), used a diamond indenter with calibrated area function before each indent set according to^[^ ^48^ ^]^ with a Cu Poisson ratio of 0.3. Remaining within the 1/10th film depth,^[^ ^49^ ^]^ 25 indents were measured per condition and averaged. Additional higher load indents (10 mN, Figure 3) exceeding this depth were performed to facilitate observation of evidence of operative deformation mechanisms by transmission electron microscopy (TEM). Nanoindentation at elevated temperatures, Figure 2B, used an in situ nanoindenter (Alemnis AG, Switzerland) in an SEM (DSM96, Zeiss, Germany), with procedures described,^[^ ^50^ ^]^ to a maximum load of 1 mN, at 0.2 mN s^‐1^, and with extracted material properties averaged over 20 indents per condition. In all cases, loading curves were corrected for thermal position drift and load cell drift, accepting only measurements with drift rates below 0.1 nm s^‐1^.

### Electrical Characterization

Electrical resistivity of the Cu films was measured using the four point probe van der Pauw method,^[^ ^51^ ^]^ noting the use of insulating Si_3_N_4_ and SiO_2_ barrier layers, and resolution of the van der Pauw formula employing a numerical solver (Matlab, Mathworks, UK).

### Statistical Analysis

All images acquired by electron microscopy have undergone, at most, brightness and contrast changes (i.e., no nonlinear adjustments) to enabled, for example, improved stitching of image series, e.g., Figure 3. Microstructural parameters were measured as follows: for twin thicknesses, average values were measured from by intercept counting over 5–7 grains in the crystal orientation described in Supporting Information S7, which covered 5–6 × 10^2^ twins per material condition. The average value quoted in Figure 1 is the average over all grains measured, weighted by the number of twins counted per grain. The error (formatted as “average ± error” in Figure 1) is the equivalently weighted standard deviation of these means, as the grain‐to‐grain variability was greater than any measurement error (e.g., ≈2 nm over ≈200–600 nm lengths measured from BF‐TEM images). Average lateral grain diameters were measured by an equivalent intercept approach, at a depth of 200–300 nm from the film surface, over at least 60 grains. W NP density was measured according to the method, sample sizes and statistical analysis reported in Supporting Information S2 and Table S1 (Supporting Information).

Hardness was calculated as an average of 25 measurements and the error bars in Figure 2 represent the standard deviation of these measurements; for hot hardness measurements, averaging was over 20 indents. The experimental section “Mechanical characterization” describes the acceptance criterion for indents.

## Conflict of Interest

The authors declare no conflict of interest.

## Author Contributions

T.E.J.E., N.R., and E.H. contributed equally to this work. T.E.J.E.: Conceptualization, Methodology, Investigation, Validation, Writing – Original Draft, Visualization, Funding acquisition; N.R.: Investigation, Validation, Writing – Original Draft, Visualization, Funding acquisition; E.H.: Investigation, Writing – Review & Editing; K.T.: Investigation, Writing – Review & Editing; B.P.: Investigation, Writing – Review & Editing; M.N.P.: Investigation, Writing – Review & Editing; X.M.: Investigation, Writing – Review & Editing, Visualization; L.P.: Methodology, Writing – Review & Editing, Supervision; J.M.: Conceptualization, Resources, Writing – Review & Editing, Supervision, Funding acquisition.

## Supporting information

Supporting Information

## Data Availability

The data that support the findings of this study are available in the Supporting Information of this article.
