# Supplementary material for: Thermally Stable Nanotwins: New Heights for Cu Mechanics
Source: Adv Sci (Weinh). 2022 Oct 26;9(34):2203544. doi: 10.1002/advs.202203544 (PMC9731721; doi:10.1002/advs.202203544)
Supplement: Supplementary file 1 — Supporting Information [file ADVS-9-2203544-s001.pdf]

ADVANCED SCIENCE
Open Access

Supporting Information

for *Adv. Sci.*, DOI 10.1002/advs.202203544

Thermally Stable Nanotwins: New Heights for Cu Mechanics

Thomas Edward James Edwards, Nadia Rohbeck, Emese Huszár, Keith Thomas, Barbara Putz, Mikhail Nikolayevich Polyakov, Xavier Maeder, Laszlo Pethö and Johann Michler*

Supporting Information

Thermally stable nanotwins: new heights for Cu mechanics

Thomas Edward James Edwards, Nadia Rohbeck, Emese Huszár, Keith Thomas, Barbara Putz, Mikhail Nikolayevich Polyakov, Xavier Maeder, Laszlo Pethö, Johann Michler*

Supplementary Material 1: Tungsten nanoparticle characterisation

High resolution high angle annular dark field scanning TEM imaging of the tungsten nanoparticles embedded in the nt-Cu film allow the A15 crystal structure to be proposed for at least a subset of the W NP: this is illustrated in Figure S1A,C,E, where the 2:1 occupancy ratio difference between specific atomic columns is seen as two levels of brightness in the HAADF images. The A15 structure is a known allotrope of tungsten[52], and is found by experiment[53] and modelling[54] to be stabilised by 6 – 22 at.% interstitial oxygen – this is consistent with the gas leak production method for the W NP, which introduces a small amount of air into the particle gun magnetron chamber to initiate particle nucleation on contaminant reaction products (likely oxides or nitrides). The lattice parameter measured from the images, 5.043 Å, is consistent with literature[52, 55].

It is evident from the multiple images of embedded W NP in Figure 1 and S1 that no consistent orientation relationship exists between the W NP and the Cu matrix (seen as background fringes of the Cu lattice planes): they are incoherent particles.

A second W NP structure appears to be captured in Figure 1 and S1B,D: however, as a result of the matrix fringes, analysis must be undertaken with caution to avoid confusion with interference (Moiré) patterns resulting from overlay of the matrix and nanoparticle lattices.

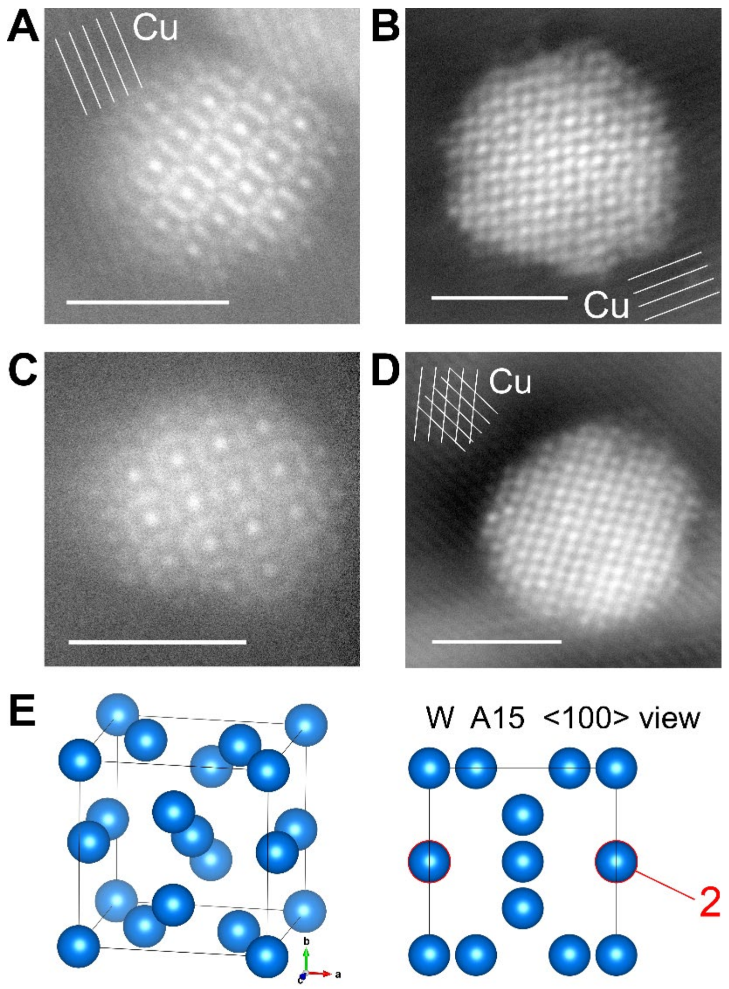

Figure S1: High resolution HAADF scanning TEM images of tungsten nanoparticles in the 0.84 vol.% containing nt-Cu film, acquired as drift correction averaged ~30 frame series. **A** (detail from Figure 1), along with **C**, is consistent with the <100> view of the A15 phase in **E**: the occupation density of the atomic columns is indicated (red ring highlighting) as multiples of unit occupation of the un-highlighted columns. The fringes of the crystal planes of the Cu matrix are annotated with white lines. The scale bars are 2 nm long.

Supplementary Material 2: W NP concentration measurements

The number density of W NP was measured by manually counting the nanoparticles on BF-STEM images of Ga^+ thinned liftout specimens across several regions per sample. A nominal 2% counting error was assumed. The Cu film volume here considered was the product of the area on the BF-STEM image times the sample thickness – the latter measured at each specific imaging and counting location by high resolution SEM using the STEM probe contamination method; a ±2 nm error was considered for each dimension, based on repeated measurement. Number density was converted to volume density knowing the average W nanoparticle diameter (4.0 nm[11]) and a conservative error in the mean of measurement by TEM of ±0.1 nm (e.g. 1 nm error per measurement from HR-TEM images, with 1174 particles measured previously[11]). Counting nanoparticles in the presence of nanotwins presented the complication of contrast from both NP and TBs; the solution was to achieve incident beam

conditions per grain which led to low contrast of the matrix/twin volumes and the TBs, see in Supplementary Material 6. For both initial W NP densities, the particle volume fraction in the films was measured in the undeformed as-deposited and 400 °C-annealed states, as well as after hot indentation (higher W NP: 25 & 400 °C; lower: 25 °C) by measuring in a region centred 100 nm below the apex of the indent. The measured values and errors are summarised in Table S1, along with the volumes considered and the particle count to demonstrate counting statistics: due to the restricted size and gradient of the plastic zone below the indent, a reduced number of particles was counted compared to the undeformed state. It should be noted that counting W NP in the deformed condition presented the additional difficulty that dislocation intersections and debris generated additional point features in bright field imaging; use of the atomic number contrast of HAADF images in combination with their BF counterparts facilitated W NP identification.

Table S1: Density of W nanoparticles as: vol.% (indent depth) [counted volume $\times 10^{-3}$ /µm^3; number of particles]. Concentrations vary minorly between the as-deposited and 400 °C conditions due to the use of neighbouring samples of the wafer (~5 mm apart) for hot hardness and annealing studies.

Sample designation	Condition	Location	
		Undeformed	Below indent
Lower W	As-deposited	0.41±0.02 [5.24; 646]	0.53±0.04 (10 mN) [0.98;155]
	400 °C	0.36±0.02 [3.38;361]	nt-Cu microstructure lost by anneal
Higher W	As-deposited	0.84±0.05 [5.78;1443]	0.85±0.06 (10 mN) [1.04;263]
	400 °C	0.97±0.06 [5.19;1496]	1.04±0.07 (1 mN) [0.92;286]

The film in Figure S2A,B is an additional deposition condition to the uniform ones in the main script: it contains variable W NP concentration through the film thickness – highest at the base, lowest in the mid-region and partway between these extremes in the upper layer. This can be achieved by several methods – varying the W magnetron power, the tolerance in size selectivity of the mass spectrometer-based device[11] and finally the power of the Cu magnetron. In the as-deposited state, the Cu microstructure is columnar – many grains are continuous from substrate to surface. For certain grains, the incident electron beam direction is appropriate to generate contrast on the horizontal nanotwins (see Supplementary Material 6). After annealing at 400 °C, this columnar structure is maintained in the upper region but is entirely lost in the middle layer: grain coarsening to a single crystal has occurred where the W

NP content was lowest, equivalently to the 0.84 vs 0.41 vol.% W NP films in the main script. Contrast in the lowest layer was too poor to assess the microstructure – due to the increased specimen thickness and higher W NP density. Room temperature spherical nanoindentation on this film to ~80% depth serves to demonstrate in Figure S2C the preferential migration of Cu, using the boundaries between differing W NP density layers as visual markers. Immediately below the indenter, the nanoparticles are seen to accumulate from the upper layer, exemplified by the atomic-number contrast in HAADF, whilst the pile-up material is considerably leaner in W NP. Although the self-diffusivity of copper is substantially greater than that of the insoluble W in the Cu matrix, at room temperature (0.22T_m) and an indentation strain rate of 0.2 s^{-1}, diffusion-based flow is not expected to control strength: any particle crowding occurs through relatively athermal crystal plasticity.

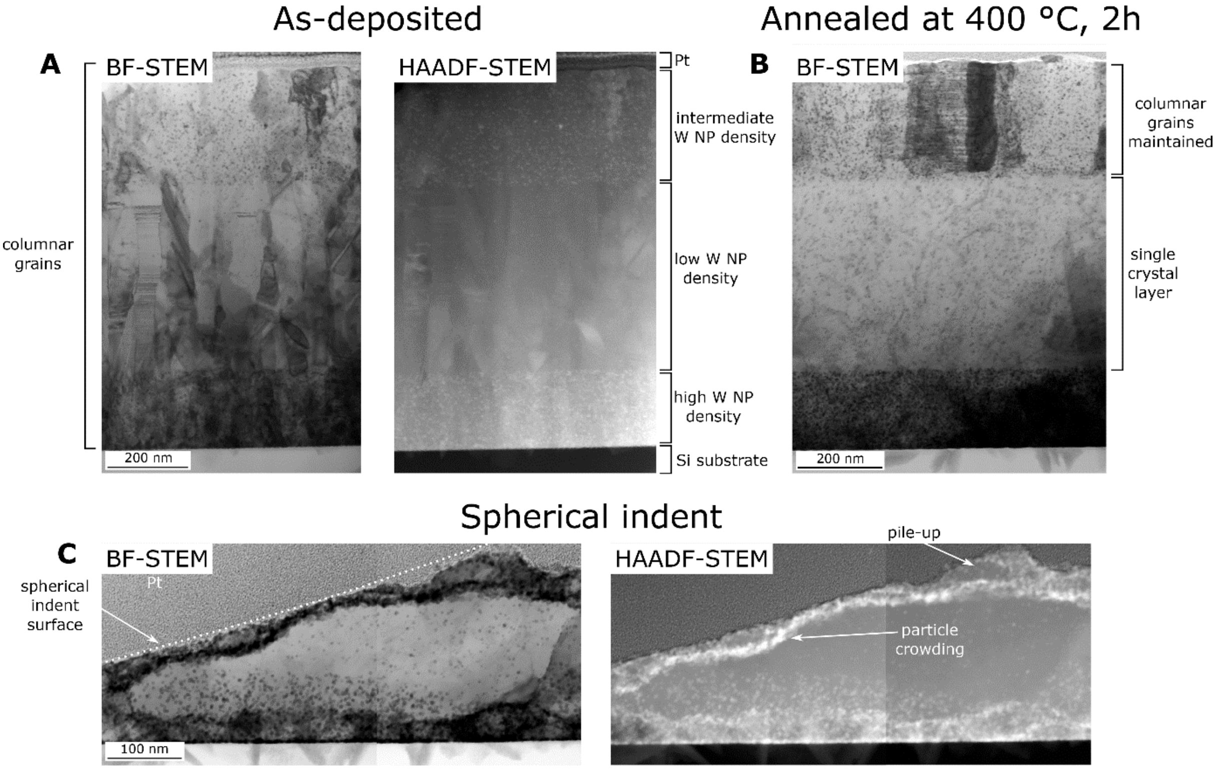

Figure S2: Scanning TEM images of a Cu film with a varying concentration of embedded W NP along the direction of growth – part adapted from[11]. (**A**) as-deposited film with columnar Cu grains – some grains are correctly orientated for nanotwin contrast (see Supplementary Material 6); (**B**) the same film after annealing at 400 °C for 2 h; the columnar grains are lost in the middle layer with the lowest W NP density. (**C**) demonstrates particle crowding in this variable W NP density film below a spherical indent.

Supplementary Material 3: Electrical resistivity analysis

The increased resistivity of nanotwinned Cu compared to oxygen-free high-conductivity copper (OFHC) (1.67×10^{-8} Ω m[14]) is known to be due to the supplementary twin boundary content and ~100 nm diameter columnar grains. Considering the 3.58×10^{-16} and 1.7×10^{-17} Ω m^2 electrical resistivity of Cu GB[56] and TB[57], respectively, the latter being

half that of an intrinsic stacking fault[58], the boundary contribution to resistivity here is 0.40 × 10^{-8} and 0.53 × 10^{-8} Ω m, respectively. Combined, these closely fulfil the 1.18 × 10^{-8} Ω m excess of the nt-Cu above the OFHC reference. Addition of W nanoparticles further raised the resistivity to 2.98 × 10^{-8} and 3.26 × 10^{-8} Ω m, for 0.41 and 0.84 vol.% W NP, respectively – only an extra 0.13 × 10^{-8} and 0.42 × 10^{-8} Ω m over the tungsten-free film due to conduction electron scattering off the nanoparticle interfaces. Hence, the Cu-Cu boundaries remain the dominant factor in raising the resistivity.

Supplementary Material 4: Additional data from indentation

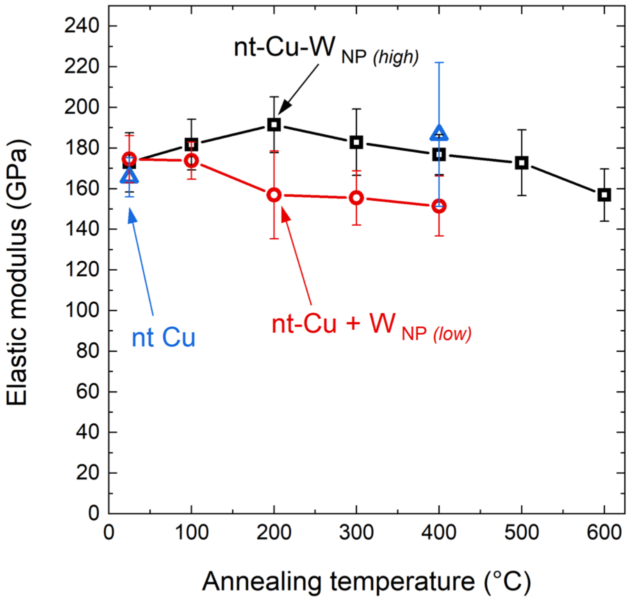

Figure S3: Room temperature indentation elastic modulus for the three material Cu film conditions (vol.% W nanoparticles) in the main script as a function of annealing temperature (1 h in vacuum per step, cumulative, except 500 and 600 °C which were each performed on separate samples as single temperature anneals) starting from the as-deposited state, as in Figure 2.

Supplementary Material 5: X-ray diffraction data

XRD data of the nt-Cu films, obtained in Bragg-Brentano geometry at room temperature following successive heat treatments, is given in Figure S4. These were analysed by peak-fitting to determine the lattice parameters and hence the thermal-induced relaxation of deposition stresses, as reported in Table S2. A ~0.2% decrease in out-of-plane lattice parameter after annealing at 200 °C is characteristic of relaxation of initial in-plane compressive stress from film deposition.

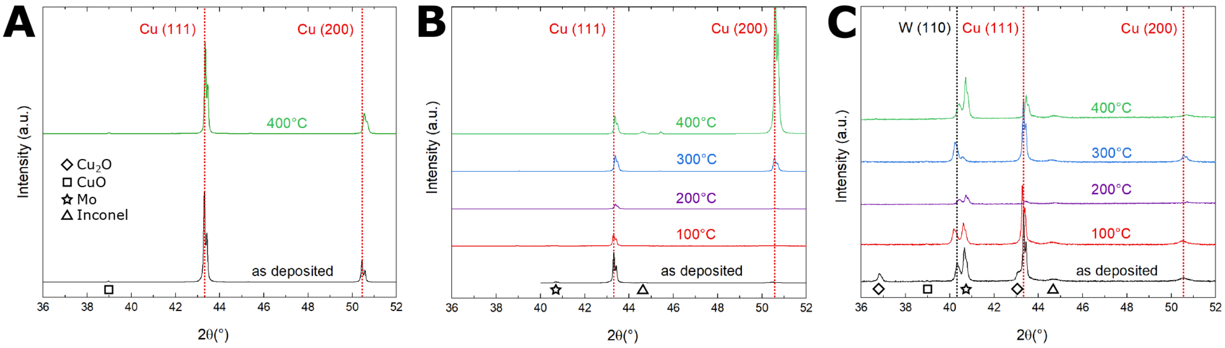

Figure S4: X-ray diffraction data of the (**A**) pure, (**B**) 0.41 vol.% W NP and (**C**) 0.84 vol.% W NP nt-Cu films, obtained in Bragg-Brentano geometry, determined a substantial change in out-of-plane texture from (111) to (200) at 300 °C (cumulative, as in Figure 2A) and above for the 0.41 vol.% W NP film, whilst the 0.84 vol.% condition revealed no significant textural evolution from (111) – additional W peaks are seen in the latter case; the Mo sample holder and Inconel fixing screws necessary for nanoindentation also occasionally give signals.

Table S2: Lattice parameter of the W nanoparticle containing films in the out-of-plane direction following annealing for 1 h.

Sample designation	Annealing temperature	Lattice parameter /Å	Change relative to initial
Low W NP	As-deposited	3.6157	N/A
	200 °C	3.6091	-0.18%
	400 °C	3.6110	-0.13%
High W NP	As-deposited	3.6138	N/A
	200 °C	3.6060	-0.22%
	400 °C	3.6044	-0.26%

Supplementary Material 6: EBSD and TEM analysis of films annealed at 500 and 600 °C

Electron backscatter diffraction analysis of the 0.84 vol.% (high) W NP content nt-Cu film after annealing for 1 h at 500 °C in Figure S5 reveals partial coarsening of the Cu grains. TEM imaging of a FIB-thinned cross-section lamella confirms this in Figure S6 and demonstrates that coarsening of the mid-depth of the film is greater than the near-surface region probed by EBSD – and most substantially contributing to the measured hardness. The coarsened grains after 1 h at 500 °C do not present a single dominant out-of-plane texture; the in-plane grain diameter average by grain area is 2.6 ± 2.0 µm, reflecting the distinct bimodality of the grain size distribution of this condition. Furthermore, the average nanotwin thickness of the retained nt-Cu grains is 6.9 ± 1.0 nm, i.e. it has coarsened from the as-deposited value of 4.1 ± 0.3 nm, or that after 1 h at 400 °C: 4.1 ± 0.5 nm.

Annealing for 1 h at 600 °C resulted in near-complete coarsening of the Cu film to a 200 dominant texture with in-plane grain diameters averaging 39 ± 19 µm (area average ± standard deviation), Figure S5.

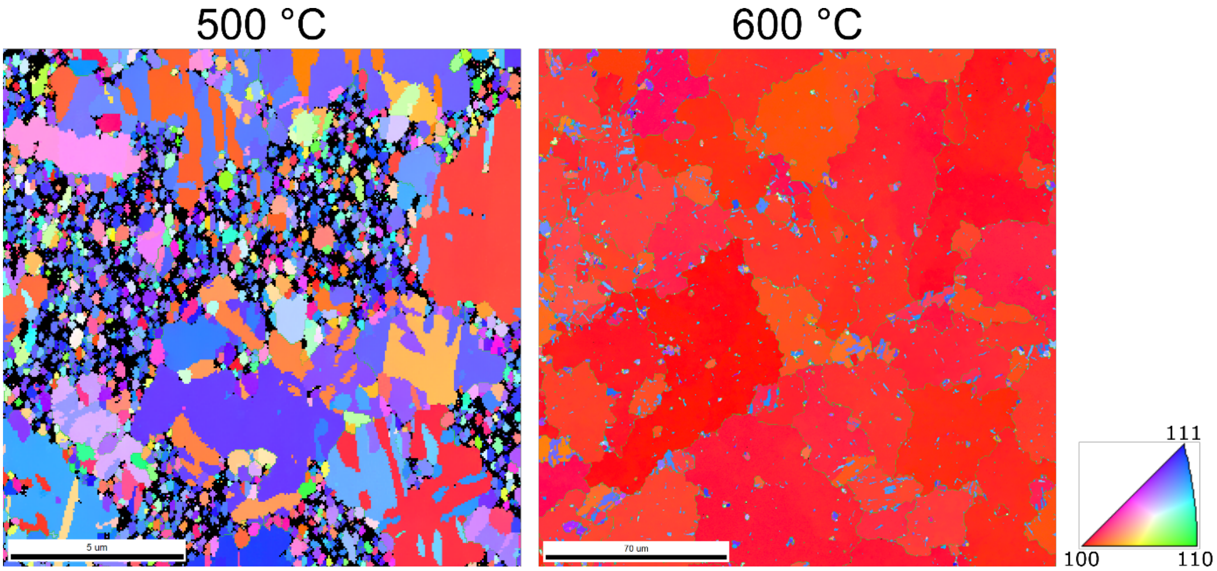

Figure S5: Electron backscatter diffraction crystal orientation maps of the 0.84 vol.% W NP containing nt-Cu film annealed at 500 and 600 °C, on separate testpieces in single temperature anneals. The inverse pole figure colour scheme is relative to the film normal direction. Both datasets have undergone a one-step grain dilation process without removal of any indexed datapoints. Black data points (generally in the retained nt-Cu regions) indicate non-indexed datapoints.

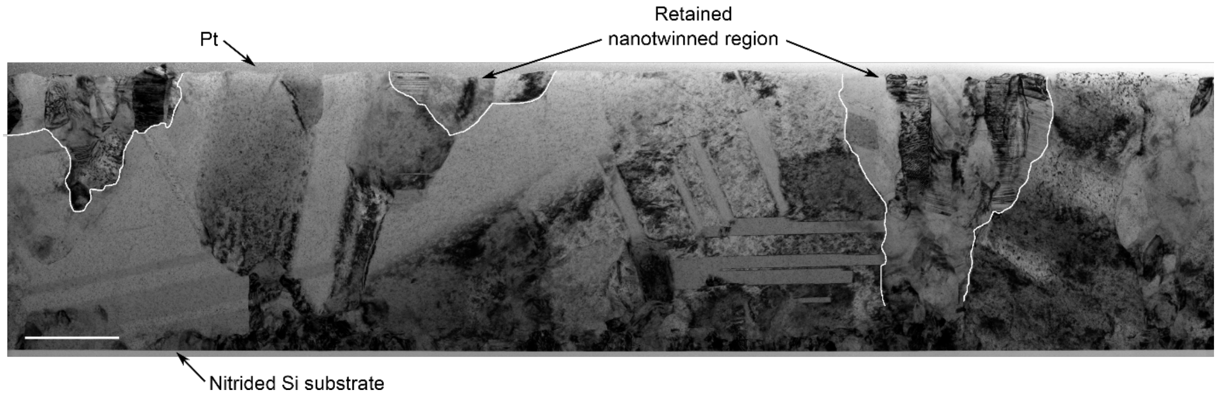

Figure S6: Bright field TEM stitched series of the high W NP nt-Cu film annealed for 1 h at 500 °C. Retained nanotwinned regions are outlined in white and are particularly located in the upper third of the film; the rest is coarse twinned, micron-scale Cu. The scale bar is 500 nm long.

Supplementary Material 7: Observation of Cu nanotwins

As detailed in Methods, the observation of nanotwins by bright field TEM is dependent on contrast conditions dictated by the incident angle of the electron beam, see example in Figure S7. In fact, the 'extinction' condition is crucial in facilitating nanoparticle counting – see Supplementary Material 2. On the other hand, the measurement of twin thicknesses is simplified when the incident beam is parallel to the coherent twin boundaries of a given grain. These considerations are important when observing the microstructure below the deep indents in Figure 3: although some of the equiaxed grains in the 0.84 & 0.41 vol.% W np film may appear twin-free at a certain viewing angle, by further tilting the sample, the twins in these grain become visible; in contrast, this is not true for those equivalent grains in the peak

plasticity zone below the indenter for the tungsten-free film where the nanotwinned microstructure is lost.

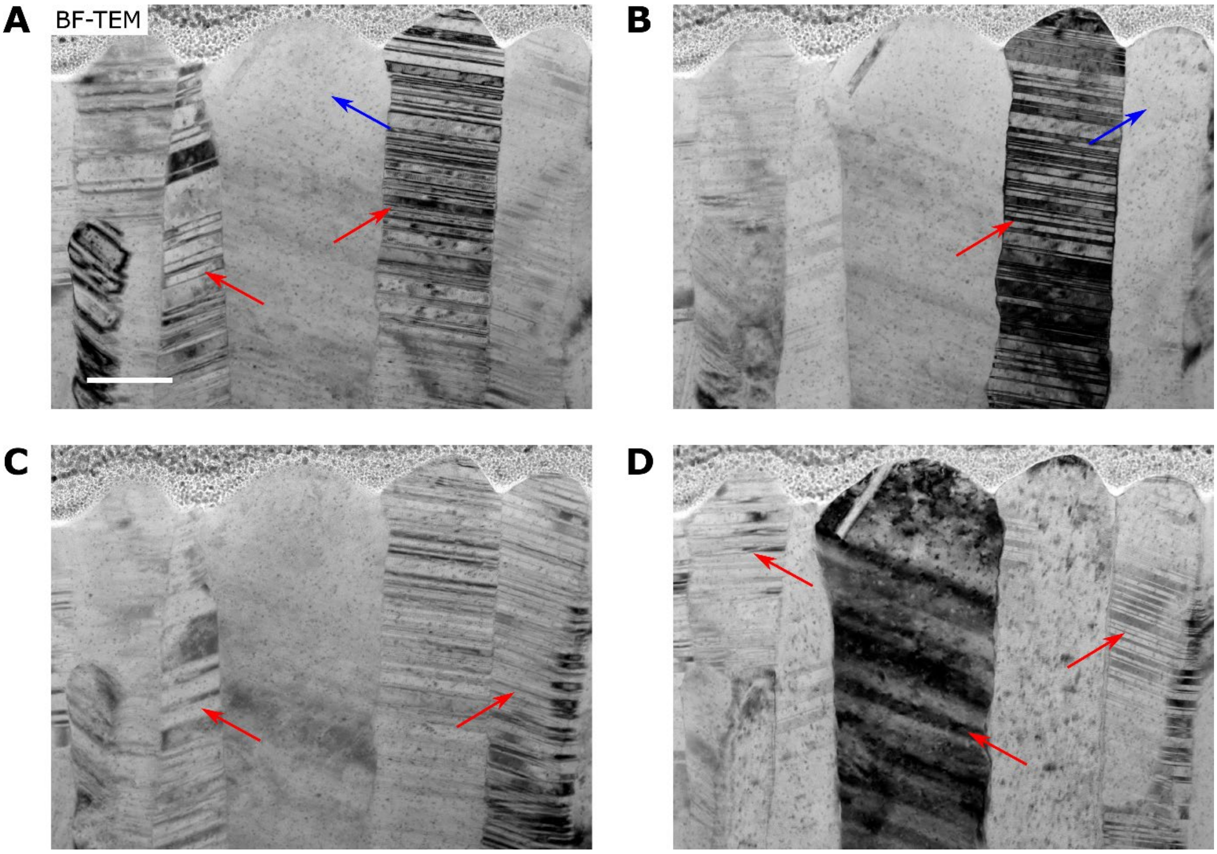

Figure S7: Illustration of incident beam orientation impact on visibility of nanotwins upon TEM bright field imaging: red arrows indicate in each image in which columns nanotwins are substantially visible, whilst blue arrows indicate appropriate imaging conditions for nanoparticle counting (nanotwin extinction). The scale bar is 100 nm long.

Supplementary Material 8: Strength/hardness calculations

For the lamellar spacing-grain size condition of the present study, the dislocation nucleation-limited regime, Equation 1, applies. A previous study[24] determined the stress concentration factor at a TB-GB intersection to be $S^* \approx 1.5/2$, which also accounts for the directionality of leading partial dislocations. The Debye frequency at room temperature is 1.3×10^{13} s^{-1} (Li *et al.*[24]), $V^* \approx 5b^3$ (within the range predicted previously[8c]) and $\Delta U \approx 1.04$ eV (Li *et al.*[24]) – the latter based on empirical data fitting[8b]. Other parameters for Cu at $T = 298$ K are given in Table S3.

A model of the cutting of dispersed particles by matrix dislocations was developed by Ansell and Lenel[59]. It considers the shear stress exerted locally by a dislocation pile-up on a second phase particle, which shears the particle at a critical stress dependent on the shear modulus of the second phase. The cutting of particles would be expected to lead to a strength increase:

$$\Delta\sigma_{\text{Cut}} = \sqrt{\frac{G b_{\text{p}} G_{\text{W}}}{2C\chi}} \qquad \text{(S1)}$$

where the shear modulus of W, G_{W}, is 164.1 GPa[60] and C is ~30 in the case of dislocation-free particles[59], which is reasonable for 4 nm W nanoparticles. Values for this stress for the materials in the present study are in Table S4.

The Hall-Petch strength is calculated as follows: TB spacing, λ, is taken as the characteristic Hall-Petch length when considerably smaller than grain size, d, giving a size-based strength:

$$\sigma_{\text{HP}} = \sigma_0 + k_{\text{HP}}/\sqrt{\lambda} \qquad \text{(S2)}$$

where σ_0 (40 MPa [61]) is the inherent resistance of the Cu lattice to dislocation motion and k_{HP} (110 MPa μm^{-1} [24, 61-62] describes sensitivity to d or λ equally well.

Further strengthening mechanisms were ignored due to the low initial dislocation density outside of the TBs from deposition, hence leading to a minimal strength increase at this scale of nanotwin thickness[63]; this microstructural refinement also suppresses dislocation pile-up and locking mechanisms[23]. Load transfer to the W NP is also minimal for phase fractions below 1 vol.% [28], and solution strength of PVD impurities is negligible (e.g. Ar <0.02 at.% [64], and although O content can range 0 – 5 at.% [65], it is minimised to close to zero[65b] by surface cleanliness, low base pressures and substrate bias[65a]).

Table S3: Parameters for Cu employed in the strength models

Quantity	Value
G, shear modulus /GPa [40]	42.1
b, Burgers vector of ordinary dislocation /nm	0.256
b_{p}, Burgers vector of leading partial dislocation /nm	0.144
v, Poisson ratio [40]	0.34

Table S4: Hardness measured on samples with varying tungsten nanoparticle content, giving the average twin thickness and the calculated hardness contribution according to Orowan, using the measured W NP concentration values in Table S1 for each material condition. *In cases where nanotwins exist/remain, the dislocation nucleation stress is the limiting factor at this twin lamella thickness, and hence the Hall Petch stress is superfluous[24]. Similarly, where nanoparticles are present, it is apparent that the stress for dislocation bowing is consistently lower than that for cutting across W NP, i.e. the latter mechanism is not favoured. **For the annealed 0.41 vol.% W NP condition, the film thickness was taken – the lateral grain size now being larger than this.

	Sample	Measured hardness (GPa)	λ (nm)	d (nm)	Hall Petch* (GPa)	Dislocation nucleation (GPa)	Fraction of W nanoparticles (vol%)	Orowan bowing (GPa)	Particle cutting* (GPa)	Quadratic sum hardness (GPa)
As-deposited	nt-Cu + $W_{NPs\ (0.84\%)}$	3.2±0.6	4.1±0.3	123±6	1.75	1.02	0.84±0.05	0.32	0.76	3.2
	nt-Cu + $W_{NPs\ (0.41\%)}$	3.3±0.5	4.1±0.7	142±3	1.76	0.99	0.41±0.02	0.22	0.63	3.0
	nt-Cu	2.7±0.2	4.2±1.2	134±2	1.73	1.01	0	-	-	3.0
	nt-Cu + $W_{NPs\ (0.84\%)}$ after 3 years		4.1±0.3	122±4						
After 400°C anneal	nt-Cu + $W_{NPs\ (0.84\%)}$	2.7±0.3	4.1±0.5	132±24	1.76	1.00	0.97±0.06	0.35	0.80	3.2
	(nt-)Cu + $W_{NPs\ (0.41\%)}$	1.6±0.3	-	1502**	0.13	-	0.36±0.02	0.20	0.61	0.72
	(nt-)Cu	1.5±0.2	-	1061	0.15	-	0	-	-	0.44

Supplementary Material 9: Creep flow stress calculations

Calculations of creep and grain boundary sliding flow stresses were based on the following models:

Coble creep[66], i.e. diffusion creep along grain boundaries:

$$\sigma_{\mathrm{Coble}} = \frac{kTd^3\dot{\varepsilon}}{47\Omega\delta_{\mathrm{B}}D_{\mathrm{B0}}}\exp\left(\frac{Q_{\mathrm{B}}}{RT}\right) \qquad \text{(S3)}$$

where Ω is the volume of a single atom, i.e. $a^3/4$, δ_B is the width of a grain boundary, taken as b, D_{B0} is the prefactor for grain boundary diffusion and Q_B is the activation energy of the same process. $\dot{\varepsilon}$ is the strain rate – corresponding to the indentation strain rate of 0.2 s^{-1} here.

Blum-Zeng creep[38, 67], i.e. diffusion creep along dislocations, considering the influence of nanocrystalline grain sizes (particularly, the high density of high angle grain boundaries) on the dislocation content within grains at any moment:

$$\sigma_{\text{Blum-Zeng}} = G\left[\frac{\pi(1-\nu)M^9}{1.2^4}\right]^{1/8} \times \alpha\left[\frac{1-c+c^3}{c^3}\right]^{1/2} \times \left[\frac{kT\dot{\varepsilon}}{G\delta_B D_{B0}}\right]^{1/8} \times \exp\left(\frac{Q_B}{8RT}\right) \times \left(\frac{d}{b}\right)^{-1/2} \quad \text{(S4)}$$

where α and c are parameters for dislocation interaction with themselves, and the GB, respectively, previously determined for this material[67].

Sliding of the columnar grain boundaries:

$$\sigma_{\text{GBS}} = \frac{M}{3}\left[L\frac{\rho_m}{M_{Cu}}\left(1-\frac{T}{T_m}\right)f_g + \left(\frac{\dot{\varepsilon}_{GBS}}{\dot{\varepsilon}_{tot}} - 1\right)\frac{kT}{b^3}\right] \quad \text{(S5)}$$

where ρ_m is the density of copper at the melt point, M_{Cu} is its molar mass, T_m is its melting temperature and b is the Burgers vector of an ordinary dislocation in Cu, such that b^3 represents the activation volume for single atomic motions. f_g is the volume fraction of grain interior, which for columnar grains as here is given by $(d-2\delta_B)/d$. The fraction of the total strain rate completed by GBS, $\dot{\varepsilon}_{GBS}/\dot{\varepsilon}_{tot}$, was given a value of 0.6 – relative to previous studies[25].

TB annihilation through dislocation motion serving to migrate TBs[26]:

$$\sigma_{\text{detwinnning}} = M\left[\frac{2.28E_0\lambda}{\pi Rh} - \frac{\gamma_{isf}}{h} + \left(\frac{\dot{\varepsilon}_{DT}}{\dot{\varepsilon}_{tot}} - 1\right)\frac{2kT}{\pi R^2 h}\right] \quad \text{(S6)}$$

where E_0 is the GB energy of the Σ3(112) incoherent twin boundary generated upon detwinning, R is the radius of the disconnection semi-loop generated at the Σ3(111) coherent twin interfaces, expressed as a fraction of the grain diameter – identified to be 11% in previous work on this material. h is the width of the coherent twin boundaries, i.e. a single (111) layer: $a/\sqrt{3}$. The fraction of the total strain rate completed by detwinning, $\dot{\varepsilon}_{DT}/\dot{\varepsilon}_{tot}$, was arbitrarily given a value of 0.5, although it should be noted that $\sigma_{\text{detwinning}}$ is not significantly sensitive to this term.

For each expression, and those in the main script, the temperature dependence of terms related to the lattice parameter, a (e.g. Burgers vectors) and the shear modulus, G (e.g. $\nu_D \propto G^{1/2}$ in Equation 1 of the main script, $\sigma_0 \propto G$ and $k_{HP} \propto G^{1/2}$) are given as follows: $G = 42.1$ (1-0.54(T-298)/1357) GPa [40] and $a = 3.62$ (1+20×10^6(T-298)) Å in the range 0 – 1000 °C [68]. Model lamellar spacings and grain diameters are taken to facilitate comparison with the datasets here, using $\lambda = 4.1$ nm and $d = 130$ nm for the nanotwinned condition, and $d = 1000$ nm for the untwinned, coarse grained annealed microstructure of the 0.41 and 0

vol.% W NP films following annealing up to 400 °C. In the latter, TB migration for detwinning is, of course, omitted from the calculations.

The complete set of curves for these models and conditions are plotted in Figure S8. The remaining parameters are given in Table S5.

Some remaining microstructural complexity above 150 °C in the without and low W NP ~1 µm-grained films may explain the higher-than-Blum-Zeng remnant strength measured experimentally (e.g. 0.58 GPa hardness for the low W NP film at 400 °C).

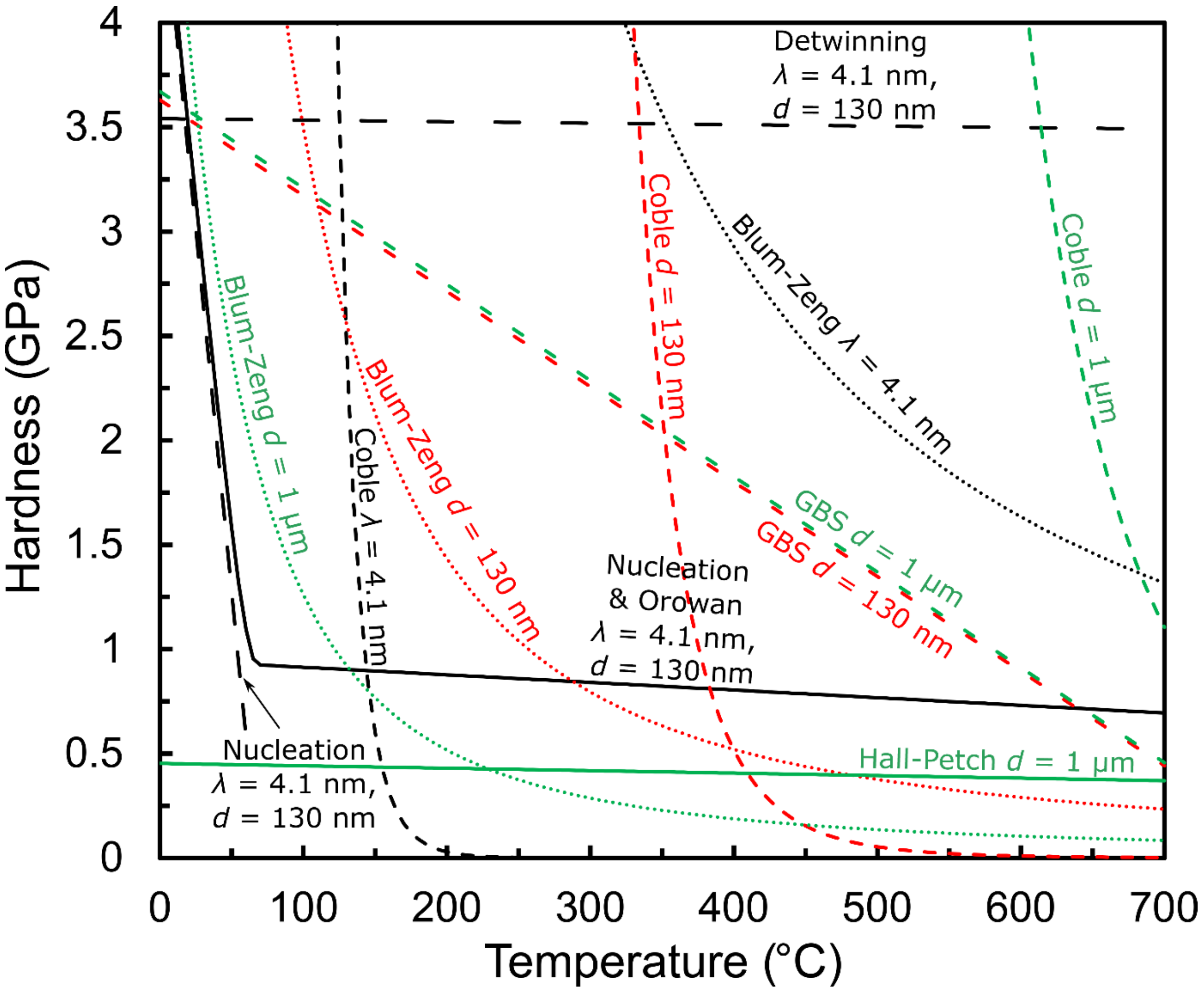

Figure S8: Models for crystal size-based strength (Hall-Petch and dislocation nucleation) and various creep mechanisms for the two model systems of $\lambda = 4.1$ nm and $d = 130$ nm for the nanotwinned condition, and $d = 1000$ nm for the untwinned, coarse grained annealed microstructure. The quadratic sum of dislocation nucleation and Orowan bowing-based strengths, as in the main script, takes the value of Orowan bowing alone at temperatures where the dislocation nucleation model would otherwise yield negative values.

Table S5: Further parameters for Cu employed in the creep and GBS models

Quantity	**Value**
a, lattice parameter Cu /nm	0.362
$\delta_B D_{B0}$, product of grain boundary width and grain boundary diffusivity pre-factor /m^2 s^{-1} [40]	5×10^{-15}
Q_B, activation energy of grain boundary diffusion /J mol^{-1} [40]	104×10^3
α, dislocation interaction parameter in Blum-Zeng creep equation [38]	0.3

c, dislocation interaction parameter in Blum-Zeng creep equation [67]	0.7
E_0, /J m^{-2} [26]	0.541
γ_{isf}, energy of intrinsic stacking fault /J m^{-2} [26]	0.032
T_m, melting temperature of Cu /K	1357
M_{Cu}, molar mass of Cu /kg mol^{-1}	64×10^{-3}
ρ_m, density of Cu at melt point /kg m^{-3} [26]	7900

Supplementary Material 10: nt-Cu thin film deposition

The cycles of the nanoparticle generator, which ensure a uniform flow rate with time of the nanoparticles by regularly supplementing the available nucleation sites, are described in Figure S1.

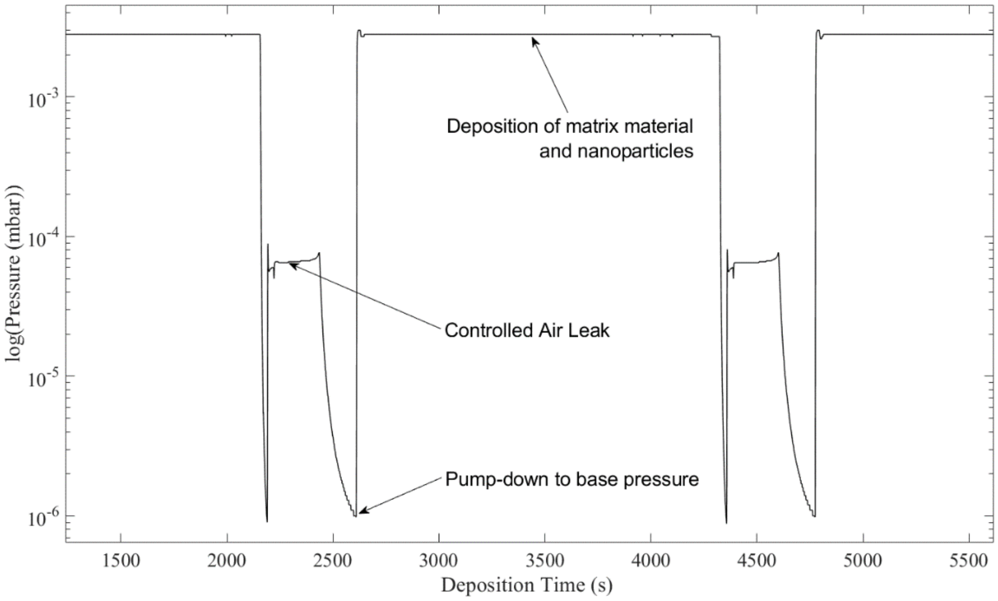

Figure S9: Pressure evolution of the main deposition chamber containing the matrix (copper) magnetron during two complete air-leak cycles.
